# Clinical application status and prospect of the combined anti-tumor strategy of ablation and immunotherapy

**DOI:** 10.3389/fimmu.2022.965120

**Published:** 2022-09-05

**Authors:** Li Yin, Xing-yu Li, Lin-lin Zhu, Gui-lai Chen, Zhuo Xiang, Qing-qing Wang, Jing-wang Bi, Qiang Wang

**Affiliations:** ^1^ Oncology Department, Shandong Second Provincial General Hospital, Jinan, China; ^2^ Key Laboratory of Marine Drugs, Ministry of Education, School of Medicine and Pharmacy, Ocean University of China, Qingdao, China

**Keywords:** cryoablation, radiofrequency ablation, microwave ablation, immunotherapy, tumor treatment

## Abstract

Image-guided tumor ablation eliminates tumor cells by physical or chemical stimulation, which shows less invasive and more precise in local tumor treatment. Tumor ablation provides a treatment option for medically inoperable patients. Currently, clinical ablation techniques are widely used in clinical practice, including cryoablation, radiofrequency ablation (RFA), and microwave ablation (MWA). Previous clinical studies indicated that ablation treatment activated immune responses besides killing tumor cells directly, such as short-term anti-tumor response, immunosuppression reduction, specific and non-specific immune enhancement, and the reduction or disappearance of distant tumor foci. However, tumor ablation transiently induced immune response. The combination of ablation and immunotherapy is expected to achieve better therapeutic results in clinical application. In this paper, we provided a summary of the principle, clinical application status, and immune effects of tumor ablation technologies for tumor treatment. Moreover, we discussed the clinical application of different combination of ablation techniques with immunotherapy and proposed possible solutions for the challenges encountered by combined therapy. It is hoped to provide a new idea and reference for the clinical application of combinate treatment of tumor ablation and immunotherapy.

## Introduction

The cancer incidence and mortality have been rising rapidly in China, with about 4.29 million new cases and 2.81 million cancer deaths in 2015 according to China National Cancer Center (NCC) statistics (1). Malignant tumor has become a major public health problem nowadays. Radiotherapy and chemotherapy is still the predominant treatment options for advanced malignant tumors after surgery nowadays (2). Most cancer patients often suffer recurrence and distant metastasis, eventually cancer-related death, although respond to initial radiotherapy and chemotherapy. With the deep research of tumor immunity, immunotherapy is a hotspot of medicine currently, represented by immune checkpoint inhibitors (ICIs), adoptive cell transfer therapy, tumor-specific vaccines and small molecule immune drugs (3). ICIs have been widely used in cancer treatment, such as anti-PD-1/PD-L1 and anti-CTLA-4 monoclonal antibodies (mAb), whereas limited therapeutic effects in those with poor PD-L1 expression, lymphocyte deficiency, immune effector cells failed to infiltrate into tumor microenvironment (TME), insufficient host recognition of tumor antigen and so on (4). A large percentage of cancer patients still benefit little form clinical treatment, with a single-agent effective rate of only 20%-40% (5). Therefore, urgent clinical needs to explore new therapeutic strategies have addressed the aforementioned therapeutic limitations.

**Figure 1:**
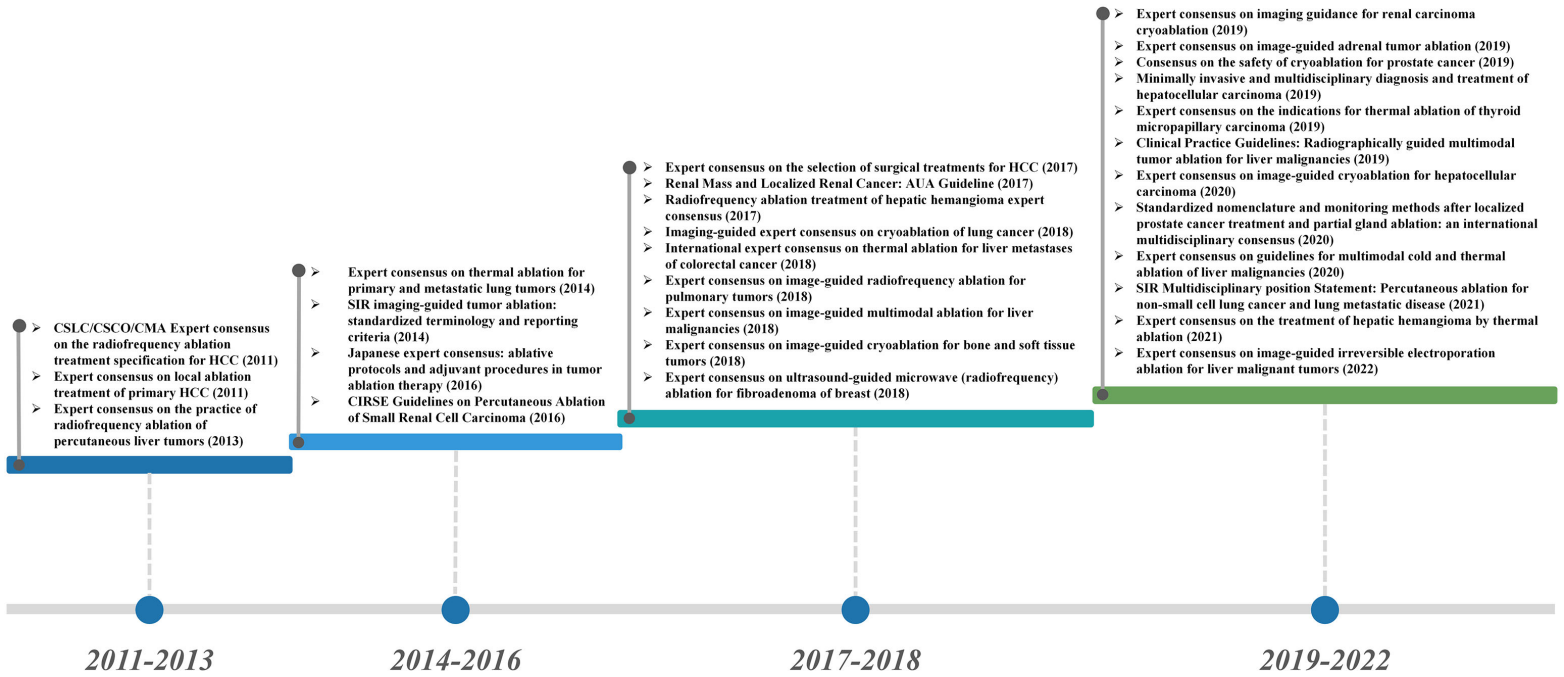
Guidelines and consensus for the application of ablation in the clinical treatment of cancer.

Ablation technology induces tumor tissue coagulative necrosis and achieves local elimination of tumor with physical and chemical methods. The ablation techniques include cryoablation, chemical ablation, laser ablation, radiofrequency ablation (RFA), microwave ablation (MWA), high-intensity focused ultrasound (HIFU), and combined technology (6). Currently, the therapeutic application of ablation technology has been widely implemented in the clinical practice, which also achieved a broad consensus and guidelines on its specific administration plans and strategies (**Figure 1**). As a minimally invasive treatment technology (except for high-intensity focused ultrasound), ablation treatment can effectively promote the exposure and release of tumor-related antigens, enhance the antigenicity of tumors, and eventually trigger a systemic antitumor immune response, which may eliminate distant metastatic lesions (7). Ablation techniques provide an optional choice for the patients with tumor oligolesion, especially those cannot withstand surgery, while potentially increasing the therapeutic effects of immunotherapy in clinical practice.

In this paper, we focused the three common used ablation technologies, including cryoablation, RFA and MWA. We reviewed the literature of ablation techniques and related immune activities, and the combination treatment of ablation and immunotherapy. We would like to provide new ideas and reference for the implementation of the combination of ablation techniques and immunotherapy.

## The principle of the ablation techniques

Imaging-guided ablation is a precise and minimally invasive cancer treatment technique, which cures tumors by physical or chemical energy safely. Currently, cryoablation, RFA and MWA are widely used in clinical practice (**Table 1**).The characteristics of tumor ablation include: ① Ablation equipment can accurately target tumor tissue with small wound, avoiding wound infection and bleeding (8–10). ② Ablation treatment shows a clear therapeutic boundary, which is generally set near the edge of tumor tissue about 1 cm (11). This range can not only effectively kill local tumor tissues, but also avoid the excessive destroy in adjacent normal tissues (12–14). ③ Ablation usually induces different degrees of autoimmune responses to inhibit tumor recurrence and metastasis (15).

**Table 1:** Selected publications on the clinical application outcomes of Cryoablation, Radiofrequency ablation (RFA), and Microwave ablation (MWA).

Modality	Tumor type	Therapeutic outcome	Reference(PMID)
Cryoablation	Hepatocellular carcinoma (HCC)	360 patients with Child-Pugh class A or B cirrhosis and one or two HCC lesions ≤ 4 cm, local tumor progression rates at 1, 2, and 3 years were 3%, 7%, and 7% for cryoablation and 9%, 11%, and 11% for RFA, respectively (P = 0.043). For lesions >3 cm in diameter, the local tumor progression rate was significantly lower in the cryoablation group versus the RFA group (7.7% versus 18.2%, P = 0.041).	25284802
	Non-small cell lung cancer (NSCLC)	Midterm survival after cryoablation is 77%-88% at 3 years in patients with early-stage NSCLC.	24991559
	Organ-confined prostate cancer	The records of 89 consecutive patients with median follow-up of 11 months (1-32) who have undergone third-generation cryosurgical ablation indicated that, at 12 months follow-up, 94% of patients achieved BDFS using ASTRO criteria while 70% achieved BDFS using a PSA threshold of < or =0.4 ng/mL.	18186694
	Extraspinal Thyroid Cancer Bone Metastases	16 patients with 18 bone metastases underwent percutaneous cryoablation (PCA) of oligometastatic extraspinal bone metastases. The 1-, 2-, 3-, 4-, and 5-year local tumor progression-free survivals were 93.3%, 84.6%, 76.9%, 75%, and 72.7%.	35318124
	Pancreatic cancer	The survival of 59 patients The median survival was 8.4 months. The overall survival at 3, 6 and 12 months was 89.7%, 61.1% and 34.5%	25083453
Radiofrequency ablation (RFA)	Recurrent thyroid cancers	15 treated lesions, 13 decreased in volume. The mean volume reduction was 50.9% (range -9.4 to 96.8%). There were gains for symptom relief for 7 patients (63.6%) The mean follow-up was 6 months (1-14 months).	21347777
	Well-differentiated thyroid cancer (DTC)	No recurrent disease was detected at the treatment site in 14 of the 16 patients treated with RFA at a mean follow-up of 40.7 months.	16858194
	Bone and soft tissue tumors	47 patients were treated with RFA. Clinical success was achieved in 94% of the patients (mean observation, 22 months). Three patients with recurrent symptoms were successfully treated with repeat RFA (secondary success rate, 100%).	11389223
	Hepatocellular carcinoma (HCC)	In the 187 patients treated with RF ablation, overall survival rates were 97% at 1 year, 71% at 3 years, and 48% at 5 years. Median survival was 57 months.	15665226
	Osteoid osteoma (OO)	The overall complication rate after RFA in the treatment of Osteoid osteoma was 3%, with skin burns being the most frequent. And the post-RFA infections being very rare.	32518986
	Uterine Fibroids	In 32 articles about 1283 patients (median age: 42 years) treated with RFA, mean procedure time about patients was 49 minutes, time to discharge was 8.2 hours, time to normal activities was 5.2 days, and time to return to work was 5.1 days. At 12 months follow-up, fibroid volume decreased by 66%, HRQL increased by 39 points, and SSS decreased by 42 points (all P <.001 versus baseline).	31702440
	Small liver colorectal metastases	Among 156 RFA ablation procedures, overall survival rates were 98.0%, 69.3%, 47.8%, 25.0%, and 18.0% (median: 53.2 months) at 1, 3, 5, 7, and 10 years. The major complication rate was 1.3% (two of 156), and there were no procedure-related deaths.	23091175
	Recurrent intrahepatic cholangiocarcinoma (ICC)	Mean local tumor progression-free survival was 39.8 months, and the cumulative local tumor progression-free 6 month and 1, 2, and 4-year survival rates were 93%, 74%, 74%, and 74%, respectively. Median overall survival after RFA was 27.4 months and the 6 month and 1, 2, and 4-year survival rates were 95%, 70%, 60%, and 21%, no procedure-related deaths.	20950977
	Isolated postsurgical local recurrences or metastases of non-small cell lung cancer (IPSLROM of NSCLC)	RFA was well tolerated by all patients. No procedure-related deaths occurred in all of the 20 ablation procedures. The overall survival rates at 1 and 2 years after RFA were 92.9% and 57.0%.	24949685
Microwave ablation (MWA)	Liver Tumors	Major complications occurred in 30 (2.6%) of 1136 patients, and these complications were immediate in four patients, periprocedural in 18 patients, and delayed in eight patients. No patients had more than two complications.	19304921
	Pancreatic tumors	The procedure was feasible in all patients (100%). Mean ablation and procedure time were respectively of 2.48 and 28 minutes. Mean hospital stay was 4 days. No major complications were observed. An improvement in QoL was observed in all patients despite a tendency to return to preoperative levels in the months following the procedure.	29770302
	Intrahepatic primary cholangiocarcinoma	The ablation success rate, the technique effectiveness rate, and the local tumor progression rate were 91.7% (22/24), 87.5% (21/24), and 25% (6/24) respectively according to the results of follow-up. The cumulative overall 6, 12, 24-month survival rates were 78.8%, 60.0%, and 60.0%.	21300500
	Desmoid fibromatosis	8 out of 9 patients (88.9%) showed improvement in the ECOG scale scores.100% reduction in the active foci was observed in 2 patients. The mean tumor volume reduction was 70.4% with a SD of 24.9% from the initial volume.	33938645
	Non-small cell lung cancer (NSCLC)	The outcomes of 35 stage I NSCLCs treated with MWA. The 1-, 2- and 3-year OS rates were 97.1%, 94.1% and 84.7%. OS and PFS for patients without local recurrence was similar to those with repeated MWA.	28449467
	Cervical metastatic lymph nodes from papillary thyroid carcinoma	All 98 metastatic lymph nodes successfully treated in a single session with 100% complete ablation. The average longest and shortest diameter of the tumors were reduced from 13.21 ± 5.86 mm to 6.74 ± 5.66 mm (p <0.01) and from 9.29 ± 4.09 mm to 4.31 ± 3.56 mm (p <0.01) at the final follow-up.	32781871

### Cryoablation

Cryoablation kills tumor cells with low temperature environment, which changes the osmotic pressure and causes tissue ischemia. The critical temperature for cell death is -20°C. Various factors influenced tumor death rate, such as freezing duration, freeze-thaw cycle and local blood flow changes (16). Cryoablation technology uses an ablation probe to introduce liquid nitrogen or argon into the tumor tissue, forming an “ice ball” near the cryoprobe, with the center temperature reaching -170°C and about 0°C in the periphery of “isotherm”. The high-pressure gas induces a freezing cycle process, in which the temperature rapidly decreases and then increases in the tumor tissue according to the Joule-Thomson principle (17). Due to the rapidly decreased temperature inside and outside cells, ice crystal leads to cell dehydration in adjacent tissues, inducing cell permeability damage and cell membrane irreversible damage, and ultimately tumor necrosis (18). Low temperature also damages the intima of microveins and arterioles in the tumor tissues. The damaged endothelial cells contact platelets to form thrombus after ice crystal thawing, which will further develop ischemia and hypoxia in the tumor tissues (19, 20).

The advantages of cryoablation are summarized as the following aspects: ① Ice crystal can be imaged by computed tomography (CT) to achieve accurate treatment area and intuitive monitoring and evaluation of the therapeutic effects during cryoablation (16). ② Cryoablation effectively reduces the recurrence rate of tumor. In SOLSTICE study, 128 post-cryoablation patients were followed up at the 12th and 24th month respectively. The local recurrence-free survival (RFS) rate after cryoablation was 85.1% and 77.2% after initial treatment. The local RFS rate of the recurrent tumor after the secondary cryoablation was even increased to 91.1% and 84.4% (21). ③Cryoablation shows significant pain alleviation effects. Previous studies indicated that cryoablation decreased the analgesics dosage in the treatment of localized tumor foci (≤4cm), which was better than RFA (22). Matthew R *et al.* summarized the most severe cancer pain assessment of 69 patients with bone metastasis before and after cryoablation, which found that the average score within 24 hours prior to cryoablation was 7.1/10, with a range of 4/10 to 10/10. The mean scores of 1, 4, 8, and 24 weeks after treatment decreased to 5.1/10 (*p*<0.001), 4.0/10 (*p <*0.001), 3.6/10 (*p <*0.001), and 1.4/10 (*p <*0.001) respectively. Cryoablation treatment provides sustain relief for bone metastatic pain (23). In a palliative treatment of bronchial cancer, cryoablation also effectively relieved the severe pain and hemoptysis which caused by bronchus blockage, and restored the bronchial ventilation again (24). Cryoablation has been widely used in the treatment of patients with early hepatocellular carcinoma (HCC), non-small cell lung cancer (NSCLC), pleural lesions, palliative treatment of lung metastatic foci, which achieved good clinical outcome (25, 26). A phase I clinical study enrolled 160 patients with NSCLC after cryoablation therapy. The average local tumor progression-free survival (PFS) interval was extended to 69 ± 2 months (27). Another study of cryoablation in recurrent pleural malignancy also showed a 3-year recurrence-free rate of local disease as 73.7% (28).

### RFA

RFA is a physical ablation that can be performed with percutaneous puncture, laparotomy or laparoscopy (29). The radiofrequency applicator is inserted into a target lesion with imaging guidance, such as ultrasound or computed tomography (CT). The current induces ions agitation at a high frequency to generate frictional heat (90-120 ℃), which causes coagulation necrosis in tumor tissues (30). Meanwhile, RFA prevents blood supply and tumor metastasis with the blood vessels disruption. A large proportion of liver tumors are suitable for RFA treatment rather than surgery resection, because of multiple intrahepatic lesions and other extrahepatic diseases (31). RFA is an optional technique for treating primary HCCs, as well as intrahepatic metastatic foci of colorectal or gastric cancer (32).

RFA exhibits significant advantages of minimal invasion, short recovery period in clinical practice. HCC patients after RFA treatment show low recurrence and mortality rate, which achieves similar curative effect as surgery to some extent (33–36). Previous studies also supported low recurrence rate and only mild adverse reactions, including fever, neutrophils, local pain and minor thermal damage in peripheral organs (37–39).

### MWA

MWA is another novel tumor ablation technology which developed from biologic thermal effects, which is mainly applied in the treatment of solid tumors in liver (40), lung (41), kidney (42), adrenal gland (43), spleen (44), thyroid (45) and breast (46). The therapeutic effects of MWA depends on the dipolar molecules (primarily water) to align and realign according to the variable electromagnetic field. The frictional heating induces tissue degeneration attributed to highly interdependent antenna–tissue interactions, which is different from RFA. So MWA is suitable for increased maximal local ablation deposition in clinical practice (47).

MWA treatment is safe and effective in local lesion treatment, which showed well tolerance for the patients. The incidence of serious complications after MWA is only 3.65%, mild complications as 8.03%, and the mortality rate is 0% according previous study (48). Both MWA and RFA are widely used in the treatment of liver tumors, while MWA is more appropriate for larger diameter liver malignancy than RFA. RFA treatment causes local tissue dehydration and carbonization to prevent heat conduction to the adjacent tumor tissues, while WMA oscillates water molecules to cause tissue heating, which expands the coagulation zone in tumor tissues (49). Medhat *et al.* evaluated 26 HCC patients with large lesions (5-7cm in diameter) with MWA treatment and found that 19/26 patients (73.1%) achieved complete ablation, while no ablation-related major complications or death (50).

## Immune effects of different ablation technologies

Tumor ablation induces immune effects by releasing immunogenic components within the tumor. Due to different ways of ablation technologies, various types and quantities of damage-associated molecular patterns (DAMPs) are released to induce different immune effects (51). Here, the differences in immune response are reviewed among different technologies.

### Immunology of cryoablation

The tumor-specific immune response caused by cryoablation is commonly known as “cryoimmune response” (52). Released tumor cell contents during ablation treatment stimulate autoimmune response, and eliminate immunosuppression effect to kill tumors. Meanwhile, the antigenicity of tumor tissue necrosis activity also stimulates immune response *in vivo* to cure distant tumor foci, which commonly known as “distal immune effect”. Previous studies have shown that tumor-specific immune responses contribute to spontaneous regression of distant metastases in patients received cryoablation treatment (53). Zhu *et al.* reported that cryoablation treatment causes DAMPs release in the center of tumor lesion by ultra-low temperature to induce tissue destruction (54), including DNA, heat shock protein (HSP), tumor antigens, cytokines and inflammatory factors (55). DAMPs phagocytosis activates NF-κB pathway to induce anti-tumor immune response of immature dendritic cells (DCs). Mature DCs translocate to lymph nodes with the help of MHC class I molecules, activating CD8^+^ T cells and costimulatory factor CD80/86 expression by presenting antigen. The immune system finally eliminates residual tumor lesions and distant metastases (56). Kato *et al.* analyzed the renal tumor samples after cryoablation treatment and found a significant increase in the proportion of T cell β receptor (TCRB) clones (defined as clones with a frequency ≥ 1% of total TCRB reads). The TCRB clones were from 27.3% ± 27.4% to 46.4% ± 31.0% after ablation (P = 0.024), the transcription levels of granase A and CD11c in CD4^+^ T cells were also significantly increased in tumor tissues (57). Other study also supported increased levels of interleukin-1 (IL-1), IL-6, NF-κB and tumor necrosis factor α (TNF-α) after cryoablation (30). Of note, IL-6 shows the most significant increase after cryoablation than RFA and MWA (58). The main reasons are summarized as follows: ① Thermal ablation causes protein denaturation in tumor tissues, and reduces the tumor antigen amounts. Meanwhile, tissue coagulation by thermal ablation reduces the release of intracellular content into circulatory system. ② Cryoablation treatment increases cell membrane permeability while maintains the intact cell structure. ③ Cryoablation increases the release of inflammatory intracellular fragments to activate immune cells, Systemic inflammatory response syndrome (SIRS) maybe observed in some serious cases, whereas rarely observed in thermal ablation technologies (30). Therefore, cryoablation showed a stronger immune response than the thermal ablation.

### Immunology of RFA

RFA technology provides a high temperature in local tumor tissue lesions for therapeutic effects. The release of large amounts of DAMPs from necrotic tissue further affect tumor microenvironment, including RNA, DNA, HSP-70, HSP-90, High Mobility Group Protein B1 (HMGB1), C-reactive protein and uric acid, which is similar to cryoablation. Zerbini *et al.* found that cell fragments released from tumor tissues after RFA contributed to the maturation of antigen-presenting cells (APCs) and DCs *in vivo*, which further promoted the activation of the immune system (59). Increased plasma levels of IL-1, IL-6, IL-8 and TNF-α were also reported after RFA treatment (60). Dramatic morphological changes were observed in tumor tissues after RFA, which were characterized as four areas: application, central, transition, and reference tissue areas (61). High temperature from RFA induces cell necrosis and increased HSP-70 expression in the transition zone (61, 62). Elevated expression levels of HSP-70 and HSP-90 are also observed in animal tumor models after RFA treatment (63). HSP-70 plays a key role in the activation of innate and adaptive immune cells (64). Ryan Slovak *et al.* indicated that innate immune response in the early stage after RFA contributed a majority part in RFA related immune response, which was associated with recurrence rate (65). Increased HSP-70 expression in tumor peripheral tissues after RFA attracts NK cells infiltration and activation, constituting the innate immunity response induced by RFA (66).

The immunosuppressive microenvironment within the tumor tissue contributes to the poor prognosis of malignancy. Among them, regulatory T cells (Tregs) induce immunosuppressive effects and mitigate the function of cytotoxic T cells (CTLs) (67). RFA treatment improves the tumor immune microenvironment by increasing the infiltration of CD4^+^ and CD8^+^T cells and reducing Tregs proportion (68, 69). The proportions of Tregs in the tumor microenvironment and blood show significantly decrease in pancreatic ductal adenocarcinoma after RFA (70). RFA treatment attenuates the immunosuppressive effects in tumor microenvironment.

### Immunology of MWA

Unlike cryoablation and RFA, no significant difference in HSP-70 expression after MWA treatment (51). However, the phenotype and number of T cells exhibit dramatic changes. Increased IL-12, IL-18 activates Inducible T Cell Co-stimulator (ICOS) pathway after MWA, which synergistically promotes Helper T cells (Th) differentiation into Th1 and increases IL-2 secretion, thus inducing the expansion of cytotoxic T cells (CTLs) related immune effect (71). Previous study showed increased percentage of T cells in peripheral blood at 1 week after MWA compared with surgery group (3.37 ± 30.31% vs. 19.42 ± 31.82%, *p*=0.033). All the studies support that WMA enhances the innate immune response (71).

The study of Zhang *et al.* observed decreased IL-4 and IL-10 levels after MWA, with IL-4 decreased from 0.13 ± 0.02 to 0.07 ± 0.01 pg/mL (p <0.05), and IL-10 decreased from 9.17 ± 2.97 to 3.93 ± 1.13 pg/mL (P <0.05) (72). So MWA inhibits Th2 cytokines secretion and reduces immunosuppressive factors in tumor microenvironment. Furthermore, ICOS expression on cell membrane after T cell activation also promotes antigen presentation of CD4 + T cells. Activated ICOS^+^ CD4^+^ T cells produce higher levels of interferon-γ (IFN-γ) (73). IFN-γ and other inflammatory factors contributes to the formation of Th1 type environment around tumor tissue, leading to immune response to eliminate tumor cells (74).

## Combinate therapy of ablation with immunotherapy

Tumor ablation technology destroys tumor tissue with minimally invasion, which produces a large amount of tumor cell fragment within the tumor lesions. The released occult antigens activate immune system, including tumor-specific T cell response. The preclinical and clinical studies indicated a remarkable increase in T cell infiltration and activation after ablation therapy. However, the ablation-induced immune response appears to be insufficient to produce sustained antitumor effects. The combination of tumor ablation and immunotherapy has been further explored in clinical applications recently, which supports the combination strategies improved ablation-induced immune effect and achieved sustained anti-tumor immune effect (**Table 2**).

**Table 2:** Overview of clinical trials of cryoablation, radiofrequency ablation(RFA) and microwave ablation (MWA) combined with immunotherapy.

Global NCT Number	Phase	Ablation type	Combination immunotherapy intervention	Type of Malignancy	Outcome Measures	References(PMID)
NCT03949153	Phase 1 Phase 2	Cryoablation	Nivolumab; Ipilimumab.	Melanoma (Skin)	Number of failures linked to the procedure.	
NCT01065441	Phase 1 Phase 2	Cryoablation	AlloStim	Solid Tumors Stage II, Stage III and Stage IV; Breast Cancer; Colorectal Cancer; Prostate Cancer; Melanoma; Ovarian Cancer; Sarcoma; Non-small Cell Lung Cancer.	The primary endpoint is the evaluation of any drug-related toxicity associated with AlloStimTM administration as well as the reversibility of such toxicity.	18834631; 18565579; 18054441.
NCT02380443	Phase 2	Cryoablation	AlloStim	Colorectal Cancer Metastatic	To determine the safety of increased frequency of dosing	23786302; 23734882; 22075702; 21123824; 18834631; 18565579; 18054441; 24777185.
NCT03546686	Phase 2	Cryoablation	Ipilimumab; Nivolumab.	Breast Cancer	Event-Free Survival	
NCT04339218	Phase 3	Cryoablation	Pembrolizumab	Lung Adenocarcinoma	1-year overall survival rate	
NCT01853618	Phase 1 Phase 2	Radiofrequency Ablation; Cryoablation	Tremelimumab	Heptocellular Cancer; Biliary Tract Neoplasms; Liver Cancer; Hepatocellular Carcinoma; Biliary Cancer.	Number of Participants with Serious and Non-Serious Adverse Events Regardless of Attribution	30578687; 30688989; 27816492; 28923358.
NCT03695835		Radiofrequency Ablation; Cryotherapy	Yervoy; Keytruda; Leukine.	Adenocarcinoma	MyVaccx immunotherapy treatment impact on late stage cancer disease	
NCT04707547	Phase 4	Radiofrequency Ablation	Nivolumab	Liver Cancer	Analysis of the number of CD8+ T	
NCT03067493	Phase 2	Radiofrequency ablation	Neo-MASCT	Primary Liver Cancer; Hepatectomy	Disease free survival	23269991; 16087270; 26933175; 14559842; 22353262.
NCT03101475	Phase 2	Radiofrequency ablation	Durvalumab; Tremelimumab	Colorectal Cancer Liver Metastases	Best overall immune response rate (iBOR) of lesions not treated by ablation/radiotherapy including the extrahepatic lesions according to iRECIST (with response confirmation)	
NCT03753659	Phase 2	Radiofrequency ablation; Microwave Ablation	Pembrolizumab	Hepatocellular Carcinoma	Objective response rate (ORR) according to RECIST 1.1	
NCT03864211	Phase 1 Phase 2	Radiofrequency ablation; microwave ablation	Toripalimab	Hepatocellular Carcinoma Non-resectable	Progression free survival	24714771; 29872177; 30805896; 30191038; 24561446;
NCT03939975	Phase 2	Radiofrequency Ablation; Microwave Ablation	Pembrolizumab; nivolumab; JS001.	Hepatocellular Carcinoma	Adverse events	33163408.
NCT04220944	Phase 1	Microwave Ablation	Sintilimab	Hepatic Carcinoma	Progression Free Survival	
NCT04805736	Phase 2	Microwave Ablation	Camrelizumab	Breast Cancer	Safety of Microwave Ablation Combined with Camrelizumab	
NCT04156087	Phase 2	Microwave Ablation	Durvalumab; Tremelimumab.	Pancreatic Cancer Non-resectable	Progression-free survival	
NCT04888806	Phase 2	Microwave Ablation	Camrelizumab	Colorectal Cancer Metastatic; Liver Metastases; Lung Metastases.	12-month progression-free survival	
NCT02851784	Phase 2 Phase 3	Microwave Ablation	adoptive immunotherapy	Hepatocellular Carcinoma	Cumulative survival rates were calculated by the Kaplan-Meier method, and comparison between Microwave Ablation and combination treatment will be done by the log-rank test	

### Cryoablation and immunotherapy

Immune checkpoint inhibitors (ICIs) achieves indelible mark in the field of tumor therapy, which exhibits remarkable safety and effects in clinical practice (75). The combination of ICIs and ablation aims to increase and prolong the antitumor immune effects. Present clinical studies usually selected the combination of anti-PD-1/PD-L1 or CTLA-4 mAb with ablation, which showed feasible safety and efficacy (76). McArthur *et al.* evaluated the safety of combinate treatment of cryoablation and Ipilimumab (CTLA-4 inhibitor) in 19 breast cancer patients. Only 1 patient suffered grade III toxicity symptoms (maculopapular rash unrelated to Ipilimumab treatment) (77). The combined treatment of cryoablation and Ipilimumab induced higher proportion of Ki67^+^ T cells and ICOS^hi^ T cells in peripheral blood than those only with cryoablation (*p*=0.05; *p*=0.005). The results suggest that combination therapy may bring on auto immune reaction and synergistic antitumor effect. A cervical cancer patient received Pembrolizumab after cryoablation, which showed a significant reduction of metastatic lesions (from 7.2 cm×6.8cm to 2.9 cm×1.5 cm) after two months treatment. Then the lesion was undetectable 3 months later and maintained complete response (CR) for 7 months (78). A retrospective study of cryotherapy combined with Pembrolizumab for liver metastatic melanoma, achieves superior efficacy to cryotherapy or immunotherapy alone. Cryotherapy improves the efficacy of anti-PD-1/PD-L1 mab in the 15 enrolled metastatic melanoma patients, with 1 patient (6.7%) achieved a complete response and 3 patients (20.0%) achieved a partial response. The objective response rate (ORR) of the combination regimen was 26.7% (95%CI 4.3-49.0%) and median PFS time was 4.0 months (95%CI 2.5-5.5) (79), which were significantly higher than only Pembrolizumab treatment (2.7 months) as Tumeh *et al.* reported (80). Moreover, grade 3-4 adverse events (AEs) were not observed in the combine treatment (79), while grade 3-5 AEs attributed to only Pembrolizumab treatment every two or three weeks occurred in 13.3%, 10.1% of advanced melanoma patients according to Caroline Robert *et al.* ‘s report (81). Cryoablation combined with Pembrolizumab is safe and effective than immunotherapy only.

DCs connects innate and acquired immune responses in tumor therapy. Toll-like receptor agonists activate and maturate DCs to uptake and present specific tumor antigens in microenvironment, which enabling acquired immune response (82). So combinate Toll-like receptor agonists with ablation technology means a promising strategy for enhancing anti-tumor immune response. Gaitanis *et al.* studied the combination treatment of cryoablation with Imiquimod (Toll-like receptor 7 agonist) for basal cell carcinoma, and only 1 patient (n = 21) suffered relapse after at least 18 months of follow-up with a cumulative efficacy of 95% (83).

Adoptive cell immunotherapy developed rapidly these years. The combination of tumor ablation and immune cell therapy is worth expecting. The metastatic pancreatic cancer patients treated with the combination of cryoablation and DCs immunotherapy showed significantly higher median survival time than those with cryoablation alone (13 vs. 7 months, *p*<0.001) *(* 84). Lin *et al.* evaluated the clinical efficacy of cryoablation combined with allogeneic NK cell immunotherapy in 60 renal cell carcinoma patients. The combination therapy group showed significantly lower CT values of tumor lesion and higher response rate than cryoablation alone group at 3 months post-treatment (85).

### RFA and immunotherapy

Preclinical studies indicated that RFA combined with ICIs significantly reduced the tumor burthen and prolong survival time in mice models (86). Further investigation also explored their clinical application. Duffy *et al.* evaluated Tremelimumab (CTLA-4 inhibitor) in combination with RFA in the patients with advanced hepatocellular carcinoma (87). Definite partial remission was achieved in 5 of 19 patients (26.3%; 95%CI:9.1-51.2%), and favorable PFS and OS estimation. The median viral load was decreased from 1275 x10^3^ IU/ml to 351 x10^3^ IU/ml in 14 patients with quantifiable data. Besides, a metastatic squamous cell lung cancer patient received Atezolizumab (PD-L1 inhibitor) treatment following RFA in left lower lung lesions. The left lung lesion showed a definite therapeutic response, while the right upper lung lesions without RFA treatment showed poor response to Atezolizumab (88). Thus, combined RFA therapy with ICIs maybe an optional regimen when the patient showed poor response to immunotherapy.

The combination of RFA and adoptive cellular immunotherapy also achieved remarkable clinical efficacy. RFA in combination with cell immunotherapy decreased the recurrence risk and disease progression estimation, as compared with RFA alone in HCC patients (recurrence rate: 72% vs. 33%). Notably, 2-year OS rates in the combination treatment and RFA alone group were 100% and 76.6%, respectively (*p*<0.05) (89). Another clinical study also found that a combination of RFA and RetroNectin activated killer (RAK) cells effectively reduced recurrent the risk of HCC, and no patients suffered recurrence at 7 months follow-up in the 7 HCC patients. The proportion of CD3^+^/CD8^+^ cells showed a continuous increase after combinate RFA and RAK treatment (36.08 ± 9.44% to 48.75 ± 9.44%, *p*=0.03), while the ratio of CD4^+^/CD8^+^ decreased (1.14 ± 0.42% to 0.65 ± 0.26%, *p*=0.02) *(* 90). Increased IFN-γ concentration in peripheral blood was observed in 5 patients (4.73 ± 2.50 pg/ml~7.64 ± 1.10 pg/mL, *p*=0.02). Zhao *et al.* followed-up HCC patients who received CIK combined with RFA after transcatheter arterial chemoembolization (TACE). The combination treatment group showed lower recurrence rate than control group (12% vs. 25.8%). Meanwhile, most patients showed decreased HBV DNA content after combined treatment, whereas only one patient decreased from 1.1×10^5^ to less than 10^3^ in the control group (91). Thus, RFA combined with CIK therapy or TACE provides an option to reduce recurrence rate of HCC and anti-HBV effect.

### MWA and immunotherapy

ICIs are still the most common option for combination treatment with MWA. A report evaluated MWA combined with Tremelimumab (CTLA-4 inhibitor) treatment in 16 refractory biliary tract cancer patients. Two patients achieved partial remission (12.5%), 5 stable disease (31.3%), and a maximum duration of 6.2 months (92). Notably, the median PFS was 3.4 months in this study, which was higher than second-line chemotherapy as previous report (2.8 months) (93). Another study of Camrelizumab (PD-1 inhibitor) with MWA for non-small cell lung cancer also achieved an ORR of 33.3% and a DCR of 61.9% (94).

The combination of adoptive cell therapy with MWA also demonstrates efficacy and safety in tumor treatment. A phase I clinical study evaluated the safety of percutaneous MWA combined with adoptive cell immunotherapy (DCs and CTLs) in HCC patients. CIK injection was performed at 5 days after MWA, DCs and CTL at 11 days after MWA. No class III/IV serious AEs was observed during the study. Decreased percentage of CD4^+^CD25^high^ Treg cells was observed after 1 month of combination therapy (2.40 ± 0.61 to 1.67 ± 0.48, *p*<0.05) and CD8^+^CD28^-^ effector cells increased significantly (11.41 ± 6.63 to 15.13 ± 7.16, *p*<0.05) *(* 95).

Staphylococcal enterotoxin C (SEC) exhibits superantigen activity to stimulate the proliferation of peripheral blood monocytes (PBMCs) and release of cytokines, such as IL-2, IFN-γ and TNF-α in a dose-dependent manner (96). Previous study indicated that SEC1 promoted the differentiation of CD4^+^ and CD8^+^ T cells to inhibit tumor growth in mice models (97). So combinate SEC with ablation is speculated to enhance antitumor effects. A phase II clinical trial evaluated MWA combined SEC intratumoral injection treatment in HCC. Totally 2000 U SEC was administrated in the edge of the tumor area after different days after MWA (24 days, 28 days, 2 months, 5 months and 7 months, respectively). Compared with the MWA treatment alone group, combined treatment group showed prolonged OS estimation (*p*=0.032), while no serious AEs associated with SEC injection (98).

## Challenges for combining ablation and immunotherapy

Accumulated clinical studies support that the combination of ablation and immunotherapy will achieve better clinical outcome than any treatment regimen alone. However, tumor ablation and immunotherapy still exhibit deficiencies and urgent problems to be solved as a new treatment strategy.

### Evaluation system for combinate ablation therapy

No unified evaluation system was established to access for the safety and efficacy of different combination strategies, in consideration of different combinations of ablation and immunotherapies. For example, RFA is considered as an effective regimen for HCC lesion less than 3cm in diameter (99). Then various combinations of RFA with anti-CTLA-4, anti-PD-1 mAb agents and DC-cytokine-induced killer (CIK) further improved a variety of survival estimation related parameters. However, we cannot provide evaluation criteria to measure the efficacy of different combination, which limits further exploration of their clinical application value.

National Health and Family Planning Commission Expert Group on tumor ablation technology management of China established an evaluation criterion in 2017, “*National restricted medical technology–Expert interpretation: Standardized management and clinical application’s quality control index of tumor ablation technology*” *(* 100), which was updated in 2021 and 2022. The evaluation criteria included a variety of operational accuracy and security index and related calculation formula, such as local lesion control after tumor ablation, serious complication rate within 30 days after tumor ablation. However, no evaluation item was provided to calculate tumor immunotherapy-related indicators, even which kinds of immune responses should be included in the efficacy evaluation system. Moreover, a reasonable evaluation system for immunotherapy efficacy should include reasonable evaluation time-point, and the correlation between immune response and clinical efficacy. Ablation-based combine immunotherapy is deficient in evaluation at present, because of a late start, few clinical studies and a lack of corresponding evaluation parameters. It is suggested to add corresponding immunotherapy evaluation parameters on the basis of the evaluation criteria in tumor ablation technology quality control. Perhaps including anti-tumor immune response indicators as other combination therapies into the efficacy evaluation system. In addition, it should be reminded that the safety assessment of ablation combination therapy is also necessary for clinical application, in view of the novel therapeutic effects and adverse reactions in clinical trials. Moreover, standardized technical and management training for the medical operators will be of great value to the combination therapy evaluation.

### Exploration in ablation combine strategies

Personalized treatment plan for cancer patient should not be ignored in the research of ablation combinate therapy. The ablation efficiency is affected by tumor size, location, ablation equipment, ablation duration and frequency in clinical practice, while immunotherapy efficiency is also affected by various factors, such as different forms and dosages in administration. Therefore, the influencing factors for ablation combine therapy are more complex, including the optimal timing of immunotherapy, the choice of adjuvant immunotherapy, and application order of ablation and immunotherapy (101). Previous study indicated that ICIs treatment after MWA, PBMCs showed higher T-cell activity than that of pre-MWA treatment (102). Besides, Imiquimod treatment after cryoablation resulted in better tumor clearance (10/10 vs 3/7 tumors; *p*=0.0147) and overall treatment efficacy (9/10 vs 2/7 relapse-free tumors; *p*=0.0345) than immunotherapy before cryoablation in basal cell carcinoma according to Georgios Gaitanis *et al. (* 103). The advanced HCC patients received intravenous NK cell therapy after cryoablation also achieved therapeutic effects (104). These results support immunotherapy after cryoablation exhibits superiority in eliminating tumors and reducing recurrence. However, more clinical trials are needed to explore the specific time and regimen of immunotherapy administration. It is necessary to continuously optimize and explore combined treatment strategies and develop accurate treatment plans.

Tumor tissue destruction caused by cryoablation depends on longer freezing time and slow thawing (105), with a maximum of 15 minutes for freezing and five minutes for thawing each cycle (106). The triple freeze-thaw protocol shows remarkable advantage in time consuming and tumor ablation zones than double freeze-thaw protocol in pulmonary ablation (107). In a study of cryoablation combinate with DC-CIK, the patients with recurrent or advanced HCC received repeated cryoablation showed longer median OS than those received single treatment (106). Treatment frequency is suggested to be an important factor for combination therapy efficiency.

Researchers also attempted to add chemotherapy and targeted therapy to the ablation combine therapy for further efficacy. Aquaporin (AQP) mediates adaptation effects associated with freezing injury, which was supported by increased AQP3 expression in tumor cells at 2 hours after freezing (108). AQP3 participates in the plasma membrane permeability and intracellular water retention, which increases the critical freezing temperature to maintain cell survival advantage with cryoablation treatment. AQP inhibitors (mercuric chloride) combinate with cryoablation significantly reverses resistance and improve treatment effects (109, 110), which supported AQP inhibitors in combination with cryoablation-immunotherapy in clinical practice. Combined cryoablation with Toripalimab (anti-PD-1) and Lenvatinib (anti-angiogenic agent) in a metastatic HCC patient also achieved CR at 7 months after treatment, and PFS as 24 months (111). Compared with cryoablation-chemotherapy and cryoablation-DC-CIK cell therapy, combinate treatment of cryoablation-immunotherapy-chemotherapy showed the longest median OS estimation in metastatic NSCLC (27 months, 95%CI, 26.6-37.0, P <0.001) (112). The combination of cryoablation, NK cell therapy and Herceptin significantly prolonged the PFS of patients with recurrent HER-2 positive breast cancer (113). Moreover, most reports of ablation combine with immunotherapy focus on cryoablation, while few studies choose MWA and RFA. Further clinical trials of MWA and RFA combination therapy are still needed to develop more accurate and efficient treatment plan.

### Adverse events in ablation combine therapy and treatment

Ablation related complications and side effects still bring troubles to clinical application despite of its relatively mild and low incidence rate. Among them, cryoshock (114), immunosuppression and thrombocytopenia (115) are observed in cryoablation. Cryoshock is a cytokine release syndrome with a mortality rate of 40% (114), which was corelated with dysfunction of suppressor T cells (116). Cryoshock usually occurs in the patients received cryoablation with a large tumor volume or simultaneous treatment in multiple areas (117). However, the mechanism of cryoablation induced immunosuppressive response is still unclear. Some researchers suggest its relation with cytokines release of TNF-α, IL-1 and IL-6, as well as regulatory T cell proliferation (118). Thrombocytopenia is associated with double cryo-cycle therapy (119). Otto Kollmar *et al.* demonstrated that aprotinin prevented platelet capture induced by cryoablation, prolonged platelet survival time (3.3 ± 0.4 vs 2.4 ± 0.2 days), and significantly reduced platelet aggregation (local platelet activity: 14.0 ± 1.7% vs 1.9 ± 1.9%; P < 0.001) (120). Moreover, cryoablation may cause skin necrosis, fracture and other complications in the treatment of bone tumors (121). Clark Chen *et al.* irrigated the adjacent tissues of the lesion with warm saline and filled the defect after resection with bone graft substitute, which could reduce the complication rate from 25% to 2.34% (122). Therefore, improving the accuracy of cryoablation, reducing the range of cryoablation, selecting appropriate ablation points, adjusting the parameter design of freeze-thaw cycle and combining other adjuvant therapies may be effective strategies to reduce adverse reactions of ablation and improve the safety of combined immunotherapy.

Excessive RFA and ICIs are associated with impaired liver function and immune-related adverse events (IrAEs) in liver, which will cause a variety of adverse reactions, such as fever and fatigue (123, 124). High dose corticosteroids are used to alleviate the adverse reactions in clinical practice (125), which will also decrease immune response to influence the curative effect. It is suggested that short-term corticosteroid therapy may reduce the associated adverse effects during ablation combined with immunotherapy. Inadequate RFA (iRFA) is an inevitable defect in RFA treatment alone group, which will facilize tumor proliferation, migration, invasion, epithelial-mesenchymal transformation and angiogenesis by affecting the transcriptional and epigenetic regulation of residual tumor cells, ultimately leading to tumor recurrence and metastasis (126, 127). It is speculated that iRFA may be an important factor in affecting the efficacy of RFA combined immunotherapy. Previous studies have shown that XL888 (HSP90 inhibitor) combined with RFA significantly reduces STAT3 expression and phosphorylation in tumor cells, promotes tumor cell apoptosis, and ameliorates adverse effects of iRFA effectively (128). In addition, RFA may induce arterioportal fistula in the treatment of HCC cases, which limited the therapeutic outcome (129). Xu *et al.* embolized the fistula between the right hepatic artery and the right portal vein with transcatheter arterial embolization (TAE) in HCC patients. AFP (alpha fetoprotein) remained at normal levels within 3 months and intrahepatic bleeding area was reduced (130), which supported that TAE can be used as a complementary therapy in ablation combine strategies.

Previous studies have shown that thermal ablation induced DAMPs release activates tumor proliferation and development related signaling pathways in the periphery of ablation site (131, 132), which will increase the recurrence risk. Erik Velez *et al.* selected IL-6, vascular endothelial growth factor (VEGF), and hepatocyte growth factor (HGF) to assess the recurrence risk after ablation. VEGF levels were significantly reduced in 20-W MWA (5-W MWA: 5952 ± 1068 pg/mL, 20-W MWA: 3915 ± 881 pg/mL, *p*<0.05) *(* 133). Therefore, high power and short term MWA treatment will contribute to a reduced recurrence risk. Optimizing the parameters of MWA will be a new way to reduce the occurrence of adverse reactions in ablation combination therapy.

## Summary and outlook

**Figure 2:**
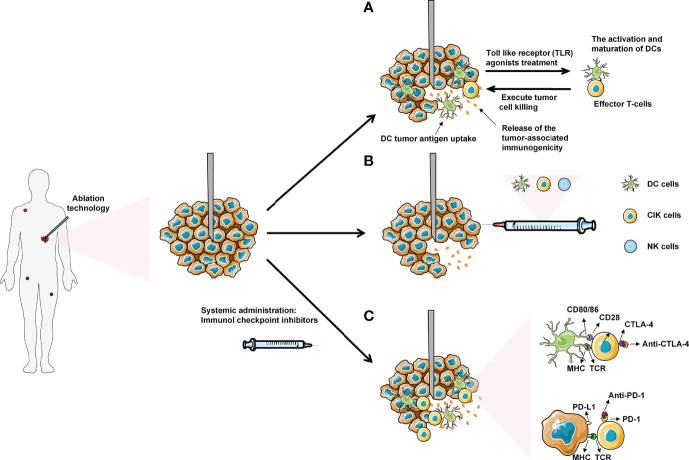
Ablation combined with systemic immunotherapies: **(A)** Administration of low dose Toll-like receptor (TLR) agonists will cause the activation and maturation of DCs. Ablation induces tumor necrosis and the release of tumor antigens into surrounding tumor microenvironment (TME), which are uptake by mature DCs in TME. The DCs present tumor antigens to naive T-cells, thereby enhancing activation and differentiation into effector T-cells to kill tumor cells. **(B)** Combined ablation with different immune cells for anti-tumor therapy. **(C)** Introduction of immune checkpoint inhibitors (anti-CTLA-4 and anti-PD-1/PDL-1) will allow the T-cells to execute tumor cell without being inhibited by the checkpoint signaling. Eventually, the effector T-cells with blocked checkpoint molecules will also migrate to the distant metastasized tumor sites, leading to the regression of metastases.

As a new local minimally invasive, safe and effective treatment method, tumor ablation technology has become a reasonable choice in the treatment of solid tumors. Ablation therapy eliminates tumor cells with physical and chemical effects. Released tumor antigen enhances anti-tumor immune response for further therapeutic effect. However, the immune response is generally non-durable. Combining ablation with immunotherapy exhibits profound synergistic effect for the treatment of tumors. In this paper, the effects of cryoablation, RFA and MWA on the immune system were thoroughly summarized. The clinical application of ablation and common immunotherapy strategy was expatiated systematically, including ICIs, small molecule immunodrugs, and adoptive cellular immunotherapy etc. (**Figure 2**).

The safety and efficacy of ablation combined immunotherapy are gradually recognized with the accumulation of relevant clinical studies. However, only a few researchers focus on the standardization of the combination regimens, resulting in the lack of a systematic analysis of literature data for therapeutic evaluation, which limits its clinical application. Therefore, it is necessary to establish a long-term evaluation system to evaluate the safety and efficacy of different combination strategies.

The combination of ablation and immunotherapy is a milestone in the history of cancer treatment. However, it is necessary to develop personalized treatment plan of combination therapy according to clinical experiences and best evidences. We should invest basic research heavily to ablation combination therapy for improved efficacy.

## Author contributions

LY and QW drafted the original manuscript. X-YL, L-LZ, G-LC, ZX, Q-QW and J-WB helped to revise the manuscript. LY and QW were responsible for leading this work and revising the manuscript. All authors listed have made a substantial, direct, and intellectual contribution to the work and approved it for publication. All authors have read and approved the final manuscript.

## Funding

This research was supported by grants from National Natural Science Foundation of China (81972793, 81803400, 81502283), Natural Science Foundation of Shandong province (ZR2020MH226, ZR2018BH045), Shandong province Medical Health Science and Technology Project (2018WS447), and Qingdao Outstanding Health Professional Development Fund.

## Acknowledgments

We apologize to those authors whose deserving research was not cited in this manuscript.

## Conflict of interest

The authors declare that the research was conducted in the absence of any commercial or financial relationships that could be construed as a potential conflict of interest.

The reviewer XY declared a shared parent affiliation with the authors LY, LZ, GC, ZX, QW, JB to the handling editor at the time of review.

## Publisher’s note

All claims expressed in this article are solely those of the authors and do not necessarily represent those of their affiliated organizations, or those of the publisher, the editors and the reviewers. Any product that may be evaluated in this article, or claim that may be made by its manufacturer, is not guaranteed or endorsed by the publisher.
